# Supplementary material for: Factors associated with shorter length of admission among people with dementia in England and Wales: retrospective cohort study
Source: BMJ Open. 2021 Oct 19;11(10):e047255. doi: 10.1136/bmjopen-2020-047255 (PMC8527135; doi:10.1136/bmjopen-2020-047255)
Supplement: Supplementary data [file bmjopen-2020-047255supp001.pdf]

Appendix 1- Predictor variables included in the analysis of factors associated with length of admission

Predictor variable	Source of data	Audit item
Type of ward	Case note audit	Q5
Primary Diagnosis	Case note audit	Q6
Discharge planning within 24 hours of admission	Case note audit	Q34
Evidence of discussing discharge with carers	Case note audit	Q29b
Executive board reviews delayed discharge	Organisational checklist	Q2b
Evidence of discussing discharge with consultant responsible for the patient	Case note audit	Q29c
Dementia specialist nurse	Organisational checklist	Q6
Social worker, or other designated person	Organisational checklist	Q34
Dementia care pathway/bundle	Organisational checklist	Q1
Liaison Hours	Liaison Psychiatry Survey of England 2	Q21
Older adult consultant	Liaison Psychiatry Survey of England 2	Q14
**Demographic variables**		
Age of the patient	Case note audit	Q1
Gender of the patient	Case note audit	Q2
Ethnicity of the patient	Case note audit	Q3

Appendix 2- List of predictor variables excluded from the analysis because over 90% of hospitals reported having implemented them.

Predictor variable	Source of data	Audit question number	Proportion of hospitals reporting them (%)
Named person/identified team with overall responsibility for complex needs discharge	Organisational checklist	Q32	95.5%
Access to intermediate care for people with dementia	Organisational checklist	Q30	92.5%

Dementia working group in place	Organisational checklist	Q9	93.5%
Evidence-based tool is used for establishing ward staffing levels	Organisational checklist	Q11	98.5%
Dementia champion at ward level	Organisational checklist	Q5a	93.5%

Appendix 3 -Number and proportion of hospitals with policies and practices in place aimed at supporting effective care for people with dementia

**Predictor variable**	**Number**	**Percentage**
Executive board reviews delayed discharge		
Yes	63	31.5%
No	137	68.5%
Dementia specialist nurse*		
Yes	64	32%
No	136	68%
Dementia care pathway/bundle		
Yes	121	60.5%
No	26	13%
In development	53	20.5%
Social worker, or other designated person		
Yes	152	76%
No	48	24%

Appendix 4 - Descriptive statistics of the predictor variables used from case note audit questionnaire (N=10106)

**Predictor variable**	**Number**	**Percentage (SD)**
**Age** (N= 10096)<br>Mean (SD)	84.3	(7.9)

**Gender**		
Male	4052	40.1%
Female	6054	59.9%
**Ethnicity**		
White	8274	81.9%
Black, Asian and Minority Ethnic	1622	16%
Not documented	210	2.1%
**Specialty of the ward**		
Cardiac	248	2.5%
Care of the elderly/complex care	4125	40.8%
Critical Care	23	0.2%
General Medical	2397	23.7%
Nephrology	52	0.5%
Obstetrics/Gynaecology	41	0.4%
Oncology	22	0.2%
Orthopaedics	906	9.0%
Stroke	457	4.5%
Surgical	686	6.8%
Other medical	1000	9.9%
Other	136	1.3%
Unknown	13	0.1%
**Primary diagnosis (N=10048)**		
Respiratory	2005	19.8
Fall	1346	13.3
Urinary/Renal	906	9.0
Hip fracture/dislocation/ other fractures/trauma	886	8.8
Sepsis	635	6.3
Delirium/Confusion/ Cognitive Impairment	1204	11.9
Gastrointestinal	595	5.9
Cardiac/ vascular / chest pain	518	5.1
Stroke + Neurological	750	7.4
Other	1239	12.3
Missing	22	0.2

Appendix 5- Number and proportion of patients receiving care according to audit standards (N=10106)

Predictor variable	Number	Percentage
**Evidence of discussing discharge with carer**		
Yes	5628	55.7%
No	1359	13.4%
N/A	398	3.9%
Missing	2721	26.9%
**Evidence of discussing discharge with consultant**		
Yes	5529	54.7%
No	1856	25.1%
Missing	2721	26.9%

**Discharge planning initiated within 24 hours of admission**		
Yes	2499	24.7%
No	2791	27.6%
N/A	2095	20.7%
Missing	2721	26.9%

Appendix 6- Provision of psychiatric liaison services at 200 hospitals

**Predictor variable**	**Number**	**Percentage**
**Liaison hours**		
No service	3	1.5%
Part-time (less than 40 hours per week)	1	0.5%
Working hours (40 hours per week)	3	1.5%
Extended Hours (More than 40 hours per week)	72	36.0%
24 Hours	91	45.5%
Missing	30	15.0%
**Team includes a specialist old age psychiatrist**		
Yes	83	41.5%
No	86	43.0%
Missing	31	15.5%

Appendix 7 -Multivariate analysis of factors associated with length of stay among 669 patients treated for hip trauma/ fracture at 170 acute hospitals

**Predictor variable**	*** Interactions**	**Estimated effect (95% CI)**	**p value**
**Ward Type** - *Care of the elderly*		-	
Orthopaedics		0.46 (0.25 to 0.66)	< 0.001
Surgical		0.34 (0.04 to 0.65)	0.29
Other medical		Ns	
Other (non-medical)		1.34 (0.31 to 2.36)	0.011
**Executive Board reviews delayed discharge**	Age		
Yes		-0.02 (-0.04 to -0.005)	0.010
No		0.01 (-0.00 to 0.20)	0.102
**Evidence of discussing discharge with carers** *Yes Vs No*		0.41 (0.27 to 0.55)	< 0.001

**Discharge planning within 24 hours of admission** *Yes Vs No*		-0.38 (-0.55 to -0.21)	< 0.001
**Ethnicity**	**Gender** *Female VS Male*		
BAME		-0.51 (-0.85 to -0.18)	0.003
White		-0.06 (-0.23 to 0.11)	0.474
**Dementia specialist nurse**	**Gender** *Female VS Male*		
Yes		-0.47 (-0.75 to -0.20)	0.001
No		-0.11 (-0.32 to 0.11)	0.33

* When an interaction effect is present the estimated effects of risk factors are interpreted taking into effect the interaction column

Appendix 8 -Multivariate analysis of factors associated with length of stay among 3375 patients treated at 74 acute hospitals with higher carer-rated satisfaction

**Predictor variable**	***Interactions**	**Estimated effect (95% CI)**	**p value**
**Ward Type -** Care of the elderly		-	
Cardiac		-0.40 (-0.64 to -0.16)	0.001
General Medical		-0.28 (-0.38 to -0.19)	< 0.001
Orthopaedics		-0.23 (-0.40 to -0.06)	0.008
Surgical		-0.39 (-0.53 to -0.25)	< 0.001
Other medical		-0.37 (-0.49 to -0.25)	< 0.001
Other (non-medical)		-0.57 (-0.86 to -0.28)	< 0.001
**Primary Diagnosis -** Respiratory		**-**	
Fall		0.30 (0.18 to 0.41)	< 0.001
Hip fracture/trauma		0.50 0.31 to 0.67	< 0.001
Cardiac/Vascular		0.21 (0.04 to 0.39)	0.019
Delirium/Confusion		0.31 (0.18 to 0.43)	< 0.001
Other		0.18 (0.05 to 0.30)	0.006
**Evidence of discussing discharge with carers**	***Gender of the patient*** *Female vs male*		

Yes		0.005<br>(-0.07 to 0.08)	0.901
No		-0.20<br>(-0.34 to -0.05)	0.008
**Discharge planning within 24 hours of admission**	**OA liaison psychiatrist consultant** *Yes vs No*		
Yes		-0.29<br>-0.46 to -0.11	0.002
No		-0.07<br>(-0.25 to 0.11)	0.425
**Social worker or other designated person**	**OA liaison psychiatrist consultant** Yes *vs No*		
Yes		-0.04<br>(-0.19 to 0.12)	0.645
No		-0.41<br>(-0.66 to -0.16)	0.002
**Evidence of discussing discharge with consultant**** *Yes vs No*		0.13<br>(0.04 to 0.22)	0.005

* When an interaction effect is present the estimated effects of risk factors are interpreted taking into effect the interaction column

** Evidence in the notes that the discharge coordinator/person or team planning discharge has discussed place of discharge and support needs with the consultant responsible for the patient's care. This can be together as a summary or recorded as separate discussions.
