# Supplementary material for: Factors associated with shorter length of admission among people with dementia in England and Wales: retrospective cohort study
Source: BMJ Open. 2021 Oct 19;11(10):e047255. doi: 10.1136/bmjopen-2020-047255 (PMC8527135; doi:10.1136/bmjopen-2020-047255)
Supplement: Author's manuscript [file bmjopen-2020-047255.draft_revisions.pdf]

BMJ Open is committed to open peer review. As part of this commitment we make the peer review history of every article we publish publicly available.

When an article is published we post the peer reviewers' comments and the authors' responses online. We also post the versions of the paper that were used during peer review. These are the versions that the peer review comments apply to.

The versions of the paper that follow are the versions that were submitted during the peer review process. They are not the versions of record or the final published versions. They should not be cited or distributed as the published version of this manuscript.

BMJ Open is an open access journal and the full, final, typeset and author-corrected version of record of the manuscript is available on our site with no access controls, subscription charges or pay-per-view fees (http://bmjopen.bmj.com).

If you have any questions on BMJ Open's open peer review process please email info.bmjopen@bmj.com

Factors associated with shorter length of admission among people with dementia in England and Wales: Retrospective cohort study.

Journal:	*BMJ Open*
Manuscript ID	bmjopen-2020-047255
Article Type:	Original research
Date Submitted by the Author:	23-Nov-2020

Complete List of Authors:	Sanatinia, Rahil; Imperial College London, Division of Psychiatry<br>Burns, Alistair; The University of Manchester, Division of Neuroscience and Experimental Psychology<br>Crome, Peter; University College London, Research Department of Primary Care and Population Health<br>Gordon, Fabiana; University of Bristol, Population Health Sciences<br>Hood, Chloe; Royal College of Psychiatrists<br>Lee, William; University of Exeter, College of Medicine and Health<br>Quirk, Alan; Royal College of Psychiatrists, CCQI<br>Seers, Kate; University of Warwick Warwick Medical School, Warwick Research in Nursing<br>Staniszewska, Sophie; University of Warwick Warwick Medical School, Warwick Research in Nursing<br>Zafarani, Gemma; Imperial College London, Division of Psychiatry<br>Crawford, Mike; Imperial College London, Division of psychiatry

Keywords:	Dementia < NEUROLOGY, Adult psychiatry < PSYCHIATRY, Quality in health care < HEALTH SERVICES ADMINISTRATION & MANAGEMENT, Organisation of health services < HEALTH SERVICES ADMINISTRATION & MANAGEMENT

SCHOLARONE™
Manuscripts

I, the Submitting Author has the right to grant and does grant on behalf of all authors of the Work (as defined in the below author licence), an exclusive licence and/or a non-exclusive licence for contributions from authors who are: i) UK Crown employees; ii) where BMJ has agreed a CC-BY licence shall apply, and/or iii) in accordance with the terms applicable for US Federal Government officers or employees acting as part of their official duties; on a worldwide, perpetual, irrevocable, royalty-free basis to BMJ Publishing Group Ltd ("BMJ") its licensees and where the relevant Journal is co-owned by BMJ to the co-owners of the Journal, to publish the Work in this journal and any other BMJ products and to exploit all rights, as set out in our licence.

The Submitting Author accepts and understands that any supply made under these terms is made by BMJ to the Submitting Author unless you are acting as an employee on behalf of your employer or a postgraduate student of an affiliated institution which is paying any applicable article publishing charge ("APC") for Open Access articles. Where the Submitting Author wishes to make the Work available on an Open Access basis (and intends to pay the relevant APC), the terms of reuse of such Open Access shall be governed by a Creative Commons licence – details of these licences and which Creative Commons licence will apply to this Work are set out in our licence referred to above.

Other than as permitted in any relevant BMJ Author's Self Archiving Policies, I confirm this Work has not been accepted for publication elsewhere, is not being considered for publication elsewhere and does not duplicate material already published. I confirm all authors consent to publication of this Work and authorise the granting of this licence.

For peer review only

Factors associated with shorter length of admission among people with dementia in England and Wales: Retrospective cohort study.

Rahil Sanatinia[1], Alistair Burns[2], Peter Crome[3], Fabiana Gordon[4], Chloe Hood[5], William Lee[6], Alan Quirk[5], Kate Seers[7], Sophie Staniszewska[7], Gemma Zafarani[1], Mike J Crawford[1,5]

1 Division of Psychiatry, Imperial College, London, UK.

2 Division of Neuroscience and Experimental Psychology, University of Manchester, Manchester, UK.

3 Research Department of Primary Care and Population Health, University College London, London, UK.

4 Population Health Sciences, University of Bristol, Bristol, UK.

5 College Centre for Quality Improvement, Royal College of Psychiatrists, London, UK.

6 College of Medicine and Health, University of Exeter, Exeter, UK.

7 Warwick Research in Nursing, Warwick Medical School, University of Warwick, Warwick, UK.

Corresponding author: Dr Rahil Sanatinia

Division of Psychiatry

Imperial College London

7th Floor Commonwealth Building, Du Cane Road

London W12 0NN

Tel - 0208 383 4161

Email: r.sanatinia@imperial.ac.uk

For peer review only

ABSTRACT

Objectives To identify aspects of the organisation and delivery of acute inpatient services for people with dementia that are associated with shorter length of hospital stay.

Design and setting Retrospective cohort study of patients admitted to 200 general hospitals in England and Wales.

Participants 10,106 people with dementia who took part in the third round of National Audit of Dementia.

Main outcome measure Length of admission to hospital

Results The median length of stay was 12 days (Inter Quartile Range = 6 to 23 days). People with dementia spent less time in hospital when discharge planning was initiated within 24 hours of admission (estimated effect -0.24, 95% confidence interval -0.29 to -0.18, p< 0.001). People from ethnic minorities had shorter length of stay (difference -0.066, 95% confidence interval -0.13 to -0.002, p=0.043). Patients with documented evidence of discussions with carers spent longer in hospital (difference 0.26, 95% confidence interval 0.21 to 0.32, p< 0.001). These associations held true in a subsample of 669 patients admitted with hip fracture and data from 74 hospitals with above average carer-rated quality of care.

Conclusions

The way that services for inpatients with dementia are delivered can influence how long they spend in hospital. Initiating discharge planning within the first 24 hours of admission may help reduce the amount of time that people with dementia spend in hospital.

For peer review only - http://bmjopen.bmj.com/site/about/guidelines.xhtml

Article Summary

Strengths and limitations of this study

- We used data from 98% of all acute hospitals in England and Wales, so our results are generalisable across the country.
- People with lived experience of dementia and caring for people with dementia helped us select items for including in the analysis and interpret the findings.
- All data were observational, so we cannot know whether the associations we found are causal
- This research relied on audit data of variable quality. Data on length of stay and organisational checklist were complete.
- We did not have data on liaison psychiatric services in a sixth of hospitals, limiting our ability to explore whether the provision of these services influences the length of stay of people with dementia

Funding statement

The work was supported by the Health Services and Delivery Research programme of the National Institute for Health Research (HS&DR 14/154/09). Infrastructure support for this research was provide by the NIHR Imperial Biomedical Research Centre (BRC).

Competing interests

Institutions for all authors have received funding from NIHR for other studies. Dr Alan Quirk and Ms Chloe Hood work at the Royal College of Psychiatrists and oversee the National Audit of Dementia. Professor Mike Crawford is Director of the College Centre for Quality Improvement at the Royal College of Psychiatrists and has been a member of the NIHR HTA General Committee. Professor Peter Crome is chair of the Steering Group for the National Audit of Dementia and has been on the HTA PCCPI Panel and HTA Prioritisation Committee A (Out of Hospital). Professor Kate Seers has been a member of the HS&DR Commissioning Board (Researcher Led). Professor Sophie Staniszewska has been on the following committees: HS&DR Associate Board Members; HS&DR Researcher Led - Associate Board Members; INVOLVE BOARD. All authors declare no other competing interests.

For peer review only - http://bmjopen.bmj.com/site/about/guidelines.xhtml

INTRODUCTION

The number of people with dementia who are admitted to acute hospitals is increasing.[1 2] The unfamiliar environment and disruption to daily routines that people with dementia experience when they are admitted to hospital can cause emotional distress and precipitate behavioural symotms.[3] When people with dementia are admitted to hospital they need more care and are more likely to suffer functional decline.[4] Patients with dementia are more impaired and vulnerable than patients without dementia and are at greater risk of adverse outcomes.[5] Compared to other inpatients, patients with dementia spend more time in hospital, are more likely to be readmitted within 28 days of their discharge,[6] and have significantly higher costs.[7 8]

Whilst specialist interventions are suggested to reduce length of stay, their impact is unclear.[9 10] Carer involvement has been identified as crucial to better patient experience during hospital admission. To achieve this, family carers need to be kept informed and provided with support and help when necessary.[11]

National audits examining acute care for people with dementia have highlighted considerable variation in the quality of care that hospitals deliver to people with dementia,[12 13] but little is known about reasons for these differences. Therefore, our objective was to identify aspects of the organisation and delivery of care for inpatients with dementia that are associated with shorter length of stay. This analysis was conducted as part of a wider mixed methods examination of factors influencing the quality of care that people with dementia receive when they are admitted to acute hospitals.[14]

METHODS

Study setting and sample

We analysed data from a retrospective cohort of patients who were enrolled in the third round of National Audit of Dementia.[12] The audit aimed to evaluate the quality of care provided by all acute hospitals in England and Wales. We used data from three components of the audit: a hospital-level organisational checklist; a retrospective case note audit with a target of a minimum of 50 sets of patient notes of patients who had been given a clinical diagnosis of dementia and been admitted to hospital for 72 hours or longer between April 2016 to November 2016; and a survey of carer experience of quality of care. We combined these with the data from the second round of national survey of Liaison Psychiatry services in England, which collected data on mental health liaison service provision in all the acute hospitals in England.[15]

For peer review only - http://bmjopen.bmj.com/site/about/guidelines.xhtml

Study measures

We calculated the length of admission for each audit participant using their date of admission and date of discharge. To select independent variables for the analysis from the wide range included in the audit, we identified factors which may influence length of stay based on a scoping review of the literature.[14] We presented this list to the members of the project management group and Stakeholder Reference Group for their feedback, including people with lived experience of dementia and carers of people with dementia. In the final stage, we excluded any variables present in or absent from over 90% of hospitals. The lists of included variables and those excluded are presented in online supplemental appendices 1 and 2.

Data management and analysis

Some of the predictor variables had multiple categories with small numbers in each category, we therefore combined these to obtain stable results [16] (details are available upon request).

We conducted univariate analyses to explore the association between the dependent variable (length of stay) and patient-level (age, gender, presenting complaint), ward-level (type of ward), and hospital-level (access to liaison mental health services, deployment of specialist dementia nurses, involvement of the Trust Board) predictor variables. We used Multiple Linear Regression for our analysis. Given the nested structure of the data, the final analysis was carried out using Hierarchical Models. All data were analysed using statistical packages STATA (version 13) and SPSS 23.

The outcome measure, length of admission, was skewed so the logarithm of length of stay was used. We excluded all those who died during their admission from all analyses.

In order to test the robustness of associations found in the main analysis, we conducted two sensitivity analyses. In the first sensitivity analysis we restricted the samples to patients who were admitted with hip fractures/ trauma. In the second sensitivity analysis we restricted the sample to patients who were admitted to hospitals that scored above the median level of carer satisfaction in the carer survey that was conducted as part of the audit.[12] Researchers who conducted the analysis of data were not involved in collecting and cleaning of data.

Ethical approval

We received approval for the secondary analysis of data from the National Clinical Audit and Patient Outcomes Programme from the Healthcare Quality Improvement Partnership prior to the start of study (Reference - HQIP162)

Patient and Public Involvement

We worked closely with the steering Group for the National Audit of Dementia, which included people with lived experience of dementia and caring for people with dementia. In addition, the

For peer review only - http://bmjopen.bmj.com/site/about/guidelines.xhtml

Project Management Group for the study included a member with dementia and a carer representative. Patients and carers contributed to the study by helping us select items for including in the analysis from the long list that we had originally developed. They also contributed to interpretation of study findings. Some of the patients involved in the early stages of the study were not able to contribute to the later stages due to the progression of their condition. One of the carer representatives (G.Z.), helped us prepare this manuscript and write the lay summary for the study.

RESULTS

In total, 200 (98.5%) of 203 acute hospitals in England and Wales took part in the audit. Organisational checklists were returned from all 200 hospitals along with data on 10,106 patient records and 4,688 carer questionnaires. The mean age of participants was 84.3 (SD=7.9), 8274 (81.9%) were White, and 6054 (59.9%) were female. Descriptive data on the predictor variables from the audit are presented in online supplemental appendices 3, 4 and 5.

Liaison Psychiatry Data

Data for the 2015 audit of psychiatric liaison services were returned by teams serving 176 (88%) of the 200 hospitals that took part in the national audit of dementia. We used data on the two predictor variables in our analysis; the number of hours covered by the liaison service, and whether the team included an old age psychiatrist. Ninety-one (53.53%) liaison services had 24-hour coverage, 72 (42.35%) were available for more than 40 hours per week, and 4 (2.35%) for less than 40 hours per week. In total, 83 of 169 (49.11%) liaison services included an old age psychiatrist. Full details can be found in online supplemental appendix 6.

Length of stay

Valid data on the length of stay were available for 10,105 patients; the median length of stay was 12 days (interquartile range = 6 to 23 days). There was variation in the median length of stay across the 200 hospitals, ranging from five to 39 days (interquartile range = 10 to 14 days).

The results of the multivariate analyses are presented in table 1. The following variables were associated with shorter average length of hospital stay: presenting with a respiratory condition, Black, Asian and Minority Ethnic background, initiating the discharge planning within 24 hours of admission, and patients for whom there was not a record of discussion with the responsible consultant (only in hospitals with a specialist old age psychiatrist). In hospitals where Trust Boards

For peer review only - http://bmjopen.bmj.com/site/about/guidelines.xhtml

For peer review only

regularly review delayed discharges, there was a non-statistically significant trend towards patients having shorter hospital stays.

Variables associated with longer average length of stay were; type of ward that patients were being treated on with those on the care of the elderly having longer average hospital stays compared to general medical and surgical wards, and discussions with carers being recorded in patients' case notes.

All risk factors were included in the initial full-effects model. This model was then reduced by excluding stepwise non-significant effects. The model presented here is the model with only the significant effects.

Table 1 - Multivariate analysis of factors associated with length of stay among 8,817 patients with dementia treated at 196 acute hospitals

**Predictor variable**	**Interactions***	**Estimated effect (95% CI)**	**p value**
**Patient age**		-0.003 (-0.005 to 2.19)	0.052
**Patient ethnicity** – White		-	
Black, Asian and Minority Ethnic		-0.066 (-0.13 to -0.002)	0.043
**Ward type** - Care of the elderly		-	
Cardiac		-0.33 (-0.47 to -0.19)	< 0.001
General Medical		-0.28 (-0.33 to -0.22)	< 0.001
Orthopaedics		-0.16 (-0.26 to -0.05)	0.003
Surgical		-0.32 (-0.41 to -0.23)	< 0.001
Other medical		-0.26 (-0.33 to -0.18)	< 0.001
Other		-0.56 (-0.74 to -0.37)	< 0.001
**Primary Diagnosis -** Respiratory		-	
Fall		0.24 (0.17 to 0.32)	< 0.001
Orthopaedic		0.39 (0.27 to 0.50)	< 0.001
Delirium/Confusion		0.25 (0.18 to 0.33)	< 0.001
Other		0.13 (0.05 to 0.20)	0.001
**Discharge planning within 24 hours of admission** *Yes Vs No*		-0.24 (-0.29 to -0.18)	< 0.001
**Executive Board reviews delayed discharge** *Yes Vs No*		-0.08 (-0.17 to 0.006)	0.069**

For peer review only - http://bmjopen.bmj.com/site/about/guidelines.xhtml

For peer review only

**Evidence of discussing discharge with carers**<br>*Yes Vs No*		0.26<br>(0.21 to 0.32)	< 0.001
**Evidence of discussing discharge with consultant**	**In hospitals with old age liaison psychiatrist**		
Yes		-0.039<br>(-0.12 to 0.05)	0.367
No		-0.15<br>(-0.03 to -0.26)	0.012

* When an interaction effect is present the estimated effects of risk factors are interpreted taking into effect the interaction column

Sensitivity analyses

Results from two sensitivity analyses are presented in online supplemental appendices (7-8). We looked at factors associated with length of stay among 669 patients treated for hip fracture at 170 acute hospitals. We also performed multivariate analysis of factors associated with length of stay among 3375 patients treated at 74 acute hospitals with higher carer-rated satisfaction. There were fewer associations in the first sensitivity analysis when we restricted the sample to patients who were admitted to hospital due to hip fracture or related injuries (n=669). The variables associated with shorter average length of stay were: documented evidence of initiating discharge planning within 24 hours of admission, Black, Asian and Minority Ethnic background (only among women but not men), and female patients in those hospitals that deployed dementia nurse specialists. When there was documented evidence that discharge had been discussed with carers, patients had longer hospital stays.

In the analysis of data from the sub-sample of patients admitted to the 74 hospitals that had carer-rated satisfaction scores above the median (n=3375), primary presenting complaint (diagnosis) and type of ward were still associated with length of stay. Patients with respiratory conditions had shorter hospital admissions compared to other diagnoses, including cardiac/vascular, fall and hip fracture. Those on the care of the elderly wards seemed to have longer lengths of stay compared to patients being treated on general medical, cardiac, orthopaedics, and surgical wards. When there was no documented evidence of discussing discharge with carers, female patients had shorter hospital admissions. In hospitals where the liaison service included an old age specialist consultant psychiatrist, patients had shorter hospital stays when there was evidence that discharge planning had been initiated within the first 24 hours of admission, as well as in hospitals where there were not any social workers or designated persons. In situations when there was evidence of discussing discharge with the consultant responsible for patient's care, average length of stay was higher. Table 2 presents a summary of results from the main multivariate and two sensitivity analyses.

For peer review only - http://bmjopen.bmj.com/site/about/guidelines.xhtml

Table 2 – Summary of results from the main multivariate and two sensitivity analyses

		Main multivariate analysis	Admitted with hip fracture (n = 669)	Admitted to hospitals with higher carer-satisfaction (n = 3375)
**Predictor variable**	**Interactions***	**Estimated effect (95% CI) P value**		
**Discharge planning within 24 hours of admission** *Yes Vs No*		-0.24 (-0.29 to -0.18) < 0.001	-0.38 (-0.55 to -0.21) < 0.001	
Yes	**Old age liaison psychiatrist consultant** ***Yes vs No***			-0.29 (-0.46 to -0.11) 0.002
No				-0.07 (-0.25 to 0.11) 0.425
**Evidence of discussing discharge with carers** *Yes Vs No*		0.26 (0.21 to 0.32) < 0.001	0.41 (0.27 to 0.55) < 0.001	
Yes	***Gender of the patient*** *Female vs male*			0.005 (-0.07 to 0.08) 0.901
No				-0.20 (-0.34 to -0.05) 0.008

For peer review only

For peer review only - http://bmjopen.bmj.com/site/about/guidelines.xhtml

DISCUSSION

We aimed to identify policies and practices that are associated with shorter hospital admissions for patients with dementia. In the multivariate analysis, in addition to presenting complaint and the type of ward that patients were admitted to, we found that the ethnicity, discharge planning within 24 hours of admission, and documented discussions about discharge with carers were associated with the amount of time that patients spent in hospital. These associations were supported by sensitivity analysis of patients admitted with a hip fracture and in hospitals with higher carer-rated quality of care for people with dementia.

Of these variables, planning discharge within 24 hours of admission had the strongest association with length of hospital stay. In a point prevalence study, researchers conducted an audit of 85 patients who spent mor than 14 days in an acute hospital in Ireland. The chart audits were completed by a clinician and a member of the multidisciplinary team. They identified timely discharge planning and clear communication as measures for preventing prolonged hospital stays. [17] Support for early discharge planning also comes from the results of a series of case studies conducted by NHS trusts across England as part of their quality improvement programmes (https://improvement.nhs.uk/resources/guide-reducing-long-hospital-stays/).

In a literature review of barriers and facilitators to discharge planning for people with dementia, involving family carers in the early stages of admission was identified to improve discharge process.[18] In our interviews with clinicians and senior managers, we found discharge planning among patients with dementia is not straightforward. Staff told us that despite the complexity, setting a planned discharge date as soon as preliminary investigations are carried out and working out the potential barriers to a timely discharge are important.[14] Senior nurses told us that allocating tasks to specific members of staff and daily review of the progress would ensure that problems are identified early and escalated to relevant departments.

In our interviews we also explored the association found between carer involvement and longer admissions. There was a consensus that involving family carers helps patients settle and adhere with treatments when admitted to hospital. Family carers usually acknowledge that long hospital stays are not conducive to patients' wellbeing. However, lack of clear communication between family carers and hospital staff from the outset may mean that expectations are not shared, which might lead to longer admissions to hospital. In a previous literature review of barriers and facilitators to discharge planning for people with dementia, researchers identified involving family carers in the early stages of admission to improve discharge process.[18]

For peer review only - http://bmjopen.bmj.com/site/about/guidelines.xhtml

The association between Black, Asian and Minority Ethnic background and shorter length of stay was unexpected and requite further investigation.

Strengths and weaknesses of the study

We used data from the third round of the National Audit of Dementia from 98% of all acute hospitals in England and Wales. This means that our results could be generalisable across the country. Data was complete on length of stay, and level of data completeness was high for key variables.

In this study we had the advantage of involvement of people with lived experience of dementia and carers of people with dementia in various stages of study planning, data analysis and interpretation. The study has several limitations. We relied on the information submitted to the audit team by hospital staff on whether hospitals had adopted a strategy or not. We cannot rule out the possibility that in some instances, certain assessments or discussions might have taken place but not been recorded. Whilst the large dataset makes the findings more generalisable, we cannot be certain if the associations we found are causal. For instance, our finding that patients whose records included documented evidence of discussions with carers having longer admissions could be causal i.e. that discussions with carers raised additional issues that they felt needed to be addressed before the person they cared for was discharged home. However, it could also be confounded by complexity and co-existing conditions of patients i.e. staff needed to liaise more with carers of patients with complex conditions, and such patients also required a longer period of treatment in hospital. It is possible that documenting discussions with carers happens more often in complex cases, where there has been more deterioration, or there is need for change of residence. [19]

There can be a trade-off between shorter hospital stay and other aspects of quality of care. Our finding that the association between shorter length of stay and early discharge planning was also present in hospitals with higher carer-rated quality of care was reassuring.

Our findings reflect practice from 2016. Since these data were collected, the COVID pandemic has further highlighted the importance of discharge planning to ensure that patient safety is maintained, and premature discharge avoided. Discharge planning at the time of COVID pandemic has been even more complicated as care homes might be unwilling to have patients back immediately after discharge from acute hospitals. Concerns about COVID outbreaks among care home residents mean that family carers are reluctant for their loved ones to return to care homes. [20 21]

Previous research has raised the possibility that psychiatric liaison services might enable more timely discharge of older adults with mental health conditions including dementia.[22 23] Missing data on

For peer review only - http://bmjopen.bmj.com/site/about/guidelines.xhtml

availability of psychiatric liaison services meant that we could not fully examine the potential impact of this factor on length of stay.

Implications for practice and future research

We recommend that hospitals put systems in place for planning discharge as soon as possible regardless of the complexity of patient presentation. Clear communication with family carers from early stages of admission helps them form realistic expectations to avoid unnecessary delays in discharge.

Future research should explore the unexpected finding of shorter hospital stays among patients with dementia of from Black Asian and Minority Ethnic backgrounds.

Contribution of authors

RS was the lead researcher, assisted with the analysis of quantitative data, and prepared the final manuscript. AB advised on the methods. PC chaired the Stakeholder Reference Group and contributed to the interpretation of the results. FG led the analysis of quantitative data. CH advised on the methods and contributed to the interpretation of the results. WL provided advice and support on the analysis of data from the second national survey of Mental Health Liaison Services. AQ contributed to the interpretation of the results. KS contributed to the interpretation of the results. SS contributed to the interpretation of the results. GZ contributed to the interpretation of the results. MJC was the chief investigator and oversaw all aspects of the study. All authors read, made comments and approved the final manuscript.

Acknowledgements

We acknowledge the valuable contribution made by our collaborators: Daphne Wallace (retired psychiatrists with lived experience), Amy Claringbold (Trial manager, Imperial College London), and Sara Hammond who led the scoping literature review and helped with the interpretation of the results.

We would also like to thank patients and carers and other members of the Stakeholder Reference Group, including: Nicci Gerrard (Carer Representative, John's Campaign), Sue Pierlejewski (Carer Representative), Hilary Doxford (Alzheimer's Society Ambassador), Jayne Goodrick (Carer Representative) Chris Roberts (Alzheimer's Society Ambassador)

For peer review only - http://bmjopen.bmj.com/site/about/guidelines.xhtml

REFERENCES

1. Ferri CP, Prince M, Brayne C, et al. Global prevalence of dementia: a Delphi consensus study. *Lancet* 2005;366(9503):2112-7. doi: 10.1016/S0140-6736(05)67889-0
2. Ombudsman HS. Care and compassion? Report of the health service ombudsman on ten investigations into NHS care of older people. London: The Stationery Office, 2011.
3. Porock D, Clissett P, Harwood RH, et al. Disruption, control and coping: responses of and to the person with dementia in hospital. *Ageing Soc* 2015;35(1):37-63. doi: 10.1017/s0144686x13000561 [published Online First: 2015/04/17]
4. Mukadam N, Sampson EL. A systematic review of the prevalence, associations and outcomes of dementia in older general hospital inpatients. *International psychogeriatrics* 2011;23(3):344-55.
5. Travers C, Byrne GJ, Pachana NA, et al. Prospective observational study of dementia in older patients admitted to acute hospitals. *Australasian journal on ageing* 2014;33(1):55-58.
6. Tropea J, LoGiudice D, Liew D, et al. Poorer outcomes and greater healthcare costs for hospitalised older people with dementia and delirium: a retrospective cohort study. *International journal of geriatric psychiatry* 2017;32(5):539-47.
7. Connolly S, O'Shea E. The impact of dementia on length of stay in acute hospitals in Ireland. *Dementia* 2015;14(5):650-58. doi: 10.1177/1471301213506922
8. Alzheimer's Society. Counting the cost. Caring for people with dementia on hospital wards. London: Alzheimer's Society 2009.
9. Griffiths P, Bridges J, Sheldon H, et al. The role of the dementia specialist nurse in acute care: a scoping review. *Journal of clinical nursing* 2015;24(9-10):1394-405. doi: 10.1111/jocn.12717
10. Goldberg SE, Bradshaw LE, Kearney FC, et al. Care in specialist medical and mental health unit compared with standard care for older people with cognitive impairment admitted to general hospital: randomised controlled trial (NIHR TEAM trial). *BMJ* 2013;347:f4132. doi: 10.1136/bmj.f4132
11. Beardon S, Patel K, Davies B, et al. Informal carers' perspectives on the delivery of acute hospital care for patients with dementia: a systematic review. *BMC geriatrics* 2018;18(1):23. doi: 10.1186/s12877-018-0710-x [published Online First: 2018/01/27]
12. Royal College of Psychiatrists. National Audit of Dementia care in general hospitals 2016-2017: Third round of audit report: Royal College of Psychiatrists, 2017.
13. Young J, Hood C, Gandesha A, et al. National Audit of Dementia care in general hospitals 2012–13: Second round audit report and update. London: Royal College of Psychiatrists, Healthcare Quality Improvement Partnership, 2013.
14. Sanatinia R, Crawford MJ, Quirk A, et al. Identifying features associated with higher-quality hospital care and shorter length of admission for people with dementia: a mixed-methods study. *Health Services and Delivery Research* 2020;8:1-92. doi: 10.3310/hsdr08220
15. Barrett J, Aitken P, Lee W. Report of the 2nd Annual Survey of Liaison Psychiatry in England. In: Liaison RotnASo, England. Pi, eds. London: National Collaborating Centre for Mental Health, 2015.
16. Barkan H. Statistics in clinical research: Important considerations. *Annals of Cardiac Anaesthesia* 2015;18(1):74.
17. Short E, D'Alton M, Dolan E, et al. 121 Patients with Prolonged Lengths of Stay. A Point Prevalence Study. *Age & Ageing* 2019;48
18. Stockwell-Smith G, Moyle W, Marshall AP, et al. Hospital discharge processes involving older adults living with dementia: An integrated literature review. *Journal of clinical nursing* 2018;27(5-6):e712-e25. doi: 10.1111/jocn.14144
19. Timmons S, O'Shea E, O'Neill D, et al. Acute hospital dementia care: results from a national audit. *BMC geriatrics* 2016;16(1):113. doi: 10.1186/s12877-016-0293-3

For peer review only - http://bmjopen.bmj.com/site/about/guidelines.xhtml

20. Mok VCT, Pendlebury S, Wong A, et al. Tackling challenges in care of Alzheimer's disease and other dementias amid the COVID-19 pandemic, now and in the future. *Alzheimers Dement* 2020:10.1002/alz.12143. doi: 10.1002/alz.12143
21. Carter R. Covid-19: The support UK care homes need to survive. *BMJ* 2020;369:m1858. doi: 10.1136/bmj.m1858
22. Singh I, Ramakrishna S, Williamson K. The Rapid Assessment Interface and Discharge service and its implications for patients with dementia. *Clinical interventions in aging* 2013;8:1101-8. doi: 10.2147/cia.S36398 [published Online First: 2013/08/30]
23. Tadros G, Salama RA, Kingston P, et al. Impact of an integrated rapid response psychiatric liaison team on quality improvement and cost savings: the Birmingham RAID model. *The Psychiatrist* 2013;37(1):4-10. doi: 10.1192/pb.bp.111.037366 [published Online First: 2018/01/02]

For peer review only

For peer review only - http://bmjopen.bmj.com/site/about/guidelines.xhtml

Appendix 1- Predictor variables included in the analysis of factors associated with length of admission

Predictor variable	Source of data	Audit item
Type of ward	Case note audit	Q5
Primary Diagnosis	Case note audit	Q6
Discharge planning within 24 hours of admission	Case note audit	Q34
Evidence of discussing discharge with carers	Case note audit	Q29b
Executive board reviews delayed discharge	Organisational checklist	Q2b
Evidence of discussing discharge with consultant responsible for the patient	Case note audit	Q29c
Dementia specialist nurse	Organisational checklist	Q6
Social worker, or other designated person	Organisational checklist	Q34
Dementia care pathway/bundle	Organisational checklist	Q1
Liaison Hours	Liaison Psychiatry Survey of England 2	Q21
Older adult consultant	Liaison Psychiatry Survey of England 2	Q14
**Demographic variables**		
Age of the patient	Case note audit	Q1
Gender of the patient	Case note audit	Q2
Ethnicity of the patient	Case note audit	Q3

For peer review only

Appendix 2- List of predictor variables excluded from the analysis because over 90% of hospitals reported having implemented them.

Predictor variable	Source of data	Audit question number	Proportion of hospitals reporting them (%)
Named person/identified team with overall responsibility for complex needs discharge	Organisational checklist	Q32	95.5%
Access to intermediate care for people with dementia	Organisational checklist	Q30	92.5%

Dementia working group in place	Organisational checklist	Q9	93.5%
Evidence-based tool is used for establishing ward staffing levels	Organisational checklist	Q11	98.5%
Dementia champion at ward level	Organisational checklist	Q5a	93.5%

Appendix 3 -Number and proportion of hospitals with policies and practices in place aimed at supporting effective care for people with dementia

**Predictor variable**	**Number**	**Percentage**
Executive board reviews delayed discharge		
Yes	63	31.5%
No	137	68.5%
Dementia specialist nurse*		
Yes	64	32%
No	136	68%
Dementia care pathway/bundle		
Yes	121	60.5%
No	26	13%
In development	53	20.5%
Social worker, or other designated person		
Yes	152	76%
No	48	24%

Appendix 4 - Descriptive statistics of the predictor variables used from case note audit questionnaire (N=10106)

**Predictor variable**	**Number**	**Percentage (SD)**
**Age** (N= 10096)<br>Mean (SD)	84.3	(7.9)

For peer review only

**Gender**		
Male	4052	40.1%
Female	6054	59.9%
**Ethnicity**		
White	8274	81.9%
Black, Asian and Minority Ethnic	1622	16%
Not documented	210	2.1%
**Specialty of the ward**		
Cardiac	248	2.5%
Care of the elderly/complex care	4125	40.8%
Critical Care	23	0.2%
General Medical	2397	23.7%
Nephrology	52	0.5%
Obstetrics/Gynaecology	41	0.4%
Oncology	22	0.2%
Orthopaedics	906	9.0%
Stroke	457	4.5%
Surgical	686	6.8%
Other medical	1000	9.9%
Other	136	1.3%
Unknown	13	0.1%
**Primary diagnosis (N=10048)**		
Respiratory	2005	19.8
Fall	1346	13.3
Urinary/Renal	906	9.0
Hip fracture/dislocation/ other fractures/trauma	886	8.8
Sepsis	635	6.3
Delirium/Confusion/ Cognitive Impairment	1204	11.9
Gastrointestinal	595	5.9
Cardiac/ vascular / chest pain	518	5.1
Stroke + Neurological	750	7.4
Other	1239	12.3
Missing	22	0.2

Appendix 5- Number and proportion of patients receiving care according to audit standards (N=10106)

Predictor variable	Number	Percentage
**Evidence of discussing discharge with carer (N=7385)**		
Yes	5628	55.7%
No	1359	13.4%
N/A	398	3.9%
Missing	2721	26.9%
**Evidence of discussing discharge with consultant (N=7385)**		
Yes	5529	54.7%
No	1856	25.1%
Missing	2721	26.9%

For peer review only

**Discharge planning initiated within 24 hours of admission (N=7385)**		
Yes	2499	24.7%
No	2791	27.6%
N/A	2095	20.7%
Missing	2721	26.9%

Appendix 6- Provision of psychiatric liaison services at 200 hospitals

**Predictor variable**	**Number**	**Percentage**
**Liaison hours**		
No service	3	1.5%
Part-time (less than 40 hours per week)	1	0.5%
Working hours (40 hours per week)	3	1.5%
Extended Hours (More than 40 hours per week)	72	36.0%
24 Hours	91	45.5%
Missing	30	15.0%
**Team includes a specialist old age psychiatrist**		
Yes	83	41.5%
No	86	43.0%
Missing	31	15.5%

For peer review only

Appendix 7 -Multivariate analysis of factors associated with length of stay among 669 patients treated for hip trauma/ fracture at 170 acute hospitals

**Predictor variable**	*** Interactions**	**Estimated effect (95% CI)**	**p value**
**Ward Type** - *Care of the elderly*		-	
Orthopaedics		0.46 (0.25 to 0.66)	< 0.001
Surgical		0.34 (0.04 to 0.65)	0.29
Other medical		Ns	
Other (non-medical)		1.34 (0.31 to 2.36)	0.011
**Executive Board reviews delayed discharge**	Age		
Yes		-0.02 (-0.04 to -0.005)	0.010
No		0.01 (-0.00 to 0.20)	0.102
**Evidence of discussing discharge with carers** *Yes Vs No*		0.41 (0.27 to 0.55)	< 0.001

**Discharge planning within 24 hours of admission** *Yes Vs No*		-0.38 (-0.55 to -0.21)	< 0.001
**Ethnicity**	**Gender** *Female VS Male*		
BAME		-0.51 (-0.85 to -0.18)	0.003
White		-0.06 (-0.23 to 0.11)	0.474
**Dementia specialist nurse**	**Gender** *Female VS Male*		
Yes		-0.47 (-0.75 to -0.20)	0.001
No		-0.11 (-0.32 to 0.11)	0.33

* When an interaction effect is present the estimated effects of risk factors are interpreted taking into effect the interaction column

Appendix 8 -Multivariate analysis of factors associated with length of stay among 3375 patients treated at 74 acute hospitals with higher carer-rated satisfaction

**Predictor variable**	***Interactions**	**Estimated effect (95% CI)**	**p value**
**Ward Type -** Care of the elderly		-	
Cardiac		-0.40 (-0.64 to -0.16)	0.001
General Medical		-0.28 (-0.38 to -0.19)	< 0.001
Orthopaedics		-0.23 (-0.40 to -0.06)	0.008
Surgical		-0.39 (-0.53 to -0.25)	< 0.001
Other medical		-0.37 (-0.49 to -0.25)	< 0.001
Other (non-medical)		-0.57 (-0.86 to -0.28)	< 0.001
**Primary Diagnosis -** Respiratory		-	
Fall		0.30 (0.18 to 0.41)	< 0.001
Hip fracture/trauma		0.50 0.31 to 0.67	< 0.001
Cardiac/Vascular		0.21 (0.04 to 0.39)	0.019
Delirium/Confusion		0.31 (0.18 to 0.43)	< 0.001
Other		0.18 (0.05 to 0.30)	0.006
**Evidence of discussing discharge with carers**	***Gender of the patient*** *Female vs male*		

For peer review only

Yes		0.005<br>(-0.07 to 0.08)	0.901
No		-0.20<br>(-0.34 to -0.05)	0.008
**Discharge planning within 24 hours of admission**	**OA liaison psychiatrist consultant** *Yes vs No*		
Yes		-0.29<br>-0.46 to -0.11	0.002
No		-0.07<br>(-0.25 to 0.11)	0.425
**Social worker or other designated person**	**OA liaison psychiatrist consultant** Yes *vs No*		
Yes		-0.04<br>(-0.19 to 0.12)	0.645
No		-0.41<br>(-0.66 to -0.16)	0.002
**Evidence of discussing discharge with consultant** *Yes vs No*		0.13<br>(0.04 to 0.22)	0.005

For peer review only

Reporting checklist for cohort study.

Based on the STROBE cohort guidelines.

Instructions to authors

Complete this checklist by entering the page numbers from your manuscript where readers will find each of the items listed below.

Your article may not currently address all the items on the checklist. Please modify your text to include the missing information. If you are certain that an item does not apply, please write "n/a" and provide a short explanation.

Upload your completed checklist as an extra file when you submit to a journal.

In your methods section, say that you used the STROBE cohortreporting guidelines, and cite them as:

von Elm E, Altman DG, Egger M, Pocock SJ, Gotzsche PC, Vandenbroucke JP. The Strengthening the Reporting of Observational Studies in Epidemiology (STROBE) Statement: guidelines for reporting observational studies.

		Reporting Item	Page Number
**Title and abstract**			
Title	#1a	Indicate the study’s design with a commonly used term in the title or the abstract	1, 2
Abstract	#1b	Provide in the abstract an informative and balanced summary of what was done and what was found	2

For peer review only

Introduction

Background / rationale	#2	Explain the scientific background and rationale for the investigation being reported	4
Objectives	#3	State specific objectives, including any prespecified hypotheses	4

Methods

Study design	#4	Present key elements of study design early in the paper	4
Setting	#5	Describe the setting, locations, and relevant dates, including periods of recruitment, exposure, follow-up, and data collection	4
Eligibility criteria	#6a	Give the eligibility criteria, and the sources and methods of selection of participants. Describe methods of follow-up.	4
Eligibility criteria	#6b	For matched studies, give matching criteria and number of exposed and unexposed	N/A
Variables	#7	Clearly define all outcomes, exposures, predictors, potential confounders, and effect modifiers. Give diagnostic criteria, if applicable	5
Data sources / measurement	#8	For each variable of interest give sources of data and details of methods of assessment (measurement). Describe comparability of assessment methods if there is more than one group. Give information separately for for	5

For peer review only

		exposed and unexposed groups if applicable.	
Bias	#9	Describe any efforts to address potential sources of bias	5
Study size	#10	Explain how the study size was arrived at	N/A
Quantitative variables	#11	Explain how quantitative variables were handled in the analyses. If applicable, describe which groupings were chosen, and why	5
Statistical methods	#12a	Describe all statistical methods, including those used to control for confounding	5
Statistical methods	#12b	Describe any methods used to examine subgroups and interactions	5
Statistical methods	#12c	Explain how missing data were addressed	5
Statistical methods	#12d	If applicable, explain how loss to follow-up was addressed	N/A
Statistical methods	#12e	Describe any sensitivity analyses	5
**Results**			
Participants	#13a	Report numbers of individuals at each stage of study—eg numbers potentially eligible, examined for eligibility, confirmed eligible, included in the study, completing follow-up, and analysed. Give information separately for for exposed and unexposed groups if applicable.	N/A, secondary analysis

For peer review only

Participants	#13b	Give reasons for non-participation at each stage	N/A
Participants	#13c	Consider use of a flow diagram	N/A
Descriptive data	#14a	Give characteristics of study participants (eg demographic, clinical, social) and information on exposures and potential confounders. Give information separately for exposed and unexposed groups if applicable.	6,and tables and appendices
Descriptive data	#14b	Indicate number of participants with missing data for each variable of interest	6
Descriptive data	#14c	Summarise follow-up time (eg, average and total amount)	N/A
Outcome data	#15	Report numbers of outcome events or summary measures over time. Give information separately for exposed and unexposed groups if applicable.	N/A
Main results	#16a	Give unadjusted estimates and, if applicable, confounder-adjusted estimates and their precision (eg, 95% confidence interval). Make clear which confounders were adjusted for and why they were included	7
Main results	#16b	Report category boundaries when continuous variables were categorized	7
Main results	#16c	If relevant, consider translating estimates of relative risk into absolute risk for a meaningful time period	7
Other analyses	#17	Report other analyses done—e.g., analyses of subgroups	8

For peer review only

		and interactions, and sensitivity analyses	
**Discussion**			
Key results	#18	Summarise key results with reference to study objectives	10
Limitations	#19	Discuss limitations of the study, taking into account sources of potential bias or imprecision. Discuss both direction and magnitude of any potential bias.	11
Interpretation	#20	Give a cautious overall interpretation considering objectives, limitations, multiplicity of analyses, results from similar studies, and other relevant evidence.	10. 11, 12
Generalisability	#21	Discuss the generalisability (external validity) of the study results	11
**Other Information**			
Funding	#22	Give the source of funding and the role of the funders for the present study and, if applicable, for the original study on which the present article is based	3

For peer review only

Notes:

- 13a: N/A, secondary analysis
- 14a: 6,and tables and appendices The STROBE checklist is distributed under the terms of the Creative Commons Attribution License CC-BY. This checklist was completed on 17. November 2020 using https://www.goodreports.org/, a tool made by the EQUATOR Network in collaboration with Penelope.ai

BMJ Open

Factors associated with shorter length of admission among people with dementia in England and Wales: Retrospective cohort study.

Journal:	*BMJ Open*
Manuscript ID	bmjopen-2020-047255.R1
Article Type:	Original research
Date Submitted by the Author:	25-May-2021
Complete List of Authors:	Sanatinia, Rahil; Imperial College London, Division of Psychiatry<br>Burns, Alistair; The University of Manchester, Division of Neuroscience and Experimental Psychology<br>Crome, Peter; University College London, Research Department of Primary Care and Population Health<br>Gordon, Fabiana; University of Bristol, Population Health Sciences<br>Hood, Chloe; Royal College of Psychiatrists<br>Lee, William; University of Exeter, College of Medicine and Health<br>Quirk, Alan; Royal College of Psychiatrists, CCQI<br>Seers, Kate; University of Warwick Warwick Medical School, Warwick Research in Nursing<br>Staniszewska, Sophie; University of Warwick Warwick Medical School, Warwick Research in Nursing<br>Zafarani, Gemma; Imperial College London, Division of Psychiatry<br>Crawford, Mike; Imperial College London, Division of psychiatry
**Primary Subject Heading**:	Health services research
Secondary Subject Heading:	Neurology, Mental health, Health services research
Keywords:	Dementia < NEUROLOGY, Adult psychiatry < PSYCHIATRY, Quality in health care < HEALTH SERVICES ADMINISTRATION & MANAGEMENT, Organisation of health services < HEALTH SERVICES ADMINISTRATION & MANAGEMENT

SCHOLARONE™
Manuscripts

I, the Submitting Author has the right to grant and does grant on behalf of all authors of the Work (as defined in the below author licence), an exclusive licence and/or a non-exclusive licence for contributions from authors who are: i) UK Crown employees; ii) where BMJ has agreed a CC-BY licence shall apply, and/or iii) in accordance with the terms applicable for US Federal Government officers or employees acting as part of their official duties; on a worldwide, perpetual, irrevocable, royalty-free basis to BMJ Publishing Group Ltd ("BMJ") its licensees and where the relevant Journal is co-owned by BMJ to the co-owners of the Journal, to publish the Work in this journal and any other BMJ products and to exploit all rights, as set out in our licence.

The Submitting Author accepts and understands that any supply made under these terms is made by BMJ to the Submitting Author unless you are acting as an employee on behalf of your employer or a postgraduate student of an affiliated institution which is paying any applicable article publishing charge ("APC") for Open Access articles. Where the Submitting Author wishes to make the Work available on an Open Access basis (and intends to pay the relevant APC), the terms of reuse of such Open Access shall be governed by a Creative Commons licence – details of these licences and which Creative Commons licence will apply to this Work are set out in our licence referred to above.

Other than as permitted in any relevant BMJ Author's Self Archiving Policies, I confirm this Work has not been accepted for publication elsewhere, is not being considered for publication elsewhere and does not duplicate material already published. I confirm all authors consent to publication of this Work and authorise the granting of this licence.

For peer review only

Factors associated with shorter length of admission among people with dementia in England and Wales*:* Retrospective cohort study.

Rahil Sanatinia[1], Alistair Burns[2], Peter Crome[3], Fabiana Gordon[4], Chloe Hood[5], William Lee[6], Alan Quirk[5], Kate Seers[7], Sophie Staniszewska[7], Gemma Zafarani[1], Mike J Crawford[1,5]

1 Division of Psychiatry, Imperial College, London, UK.

2 Division of Neuroscience and Experimental Psychology, University of Manchester, Manchester, UK.

3 Research Department of Primary Care and Population Health, University College London, London, UK.

4 Population Health Sciences, University of Bristol, Bristol, UK.

5 College Centre for Quality Improvement, Royal College of Psychiatrists, London, UK.

6 College of Medicine and Health, University of Exeter, Exeter, UK.

7 Warwick Research in Nursing, Warwick Medical School, University of Warwick, Warwick, UK.

Corresponding author: Dr Rahil Sanatinia

Division of Psychiatry

Imperial College London

2nd Floor Commonwealth Building, Du Cane Road

London W12 0NN

Tel - 0208 383 4161

Email: r.sanatinia@imperial.ac.uk

For peer review only

ABSTRACT

Objectives To identify aspects of the organisation and delivery of acute inpatient services for people with dementia that are associated with shorter length of hospital stay.

Design and setting Retrospective cohort study of patients admitted to 200 general hospitals in England and Wales.

Participants 10,106 people with dementia who took part in the third round of National Audit of Dementia.

Main outcome measure Length of admission to hospital

Results The median length of stay was 12 days (Inter Quartile Range = 6 to 23 days). People with dementia spent less time in hospital when discharge planning was initiated within 24 hours of admission (estimated effect -0.24, 95% confidence interval -0.29 to -0.18, p< 0.001). People from ethnic minorities had shorter length of stay (difference -0.066, 95% confidence interval -0.13 to -0.002, p=0.043). Patients with documented evidence of discussions having taken place between their carers and medical staff spent longer in hospital (difference 0.26, 95% confidence interval 0.21 to 0.32, p< 0.001). These associations held true in a subsample of 669 patients admitted with hip fracture and data from 74 hospitals with above average carer-rated quality of care.

Conclusions

The way that services for inpatients with dementia are delivered can influence how long they spend in hospital. Initiating discharge planning within the first 24 hours of admission may help reduce the amount of time that people with dementia spend in hospital.

For peer review only

Data availability statement

All available data can be obtained by contacting the corresponding author. All data requests should be submitted to the corresponding author for consideration. Access to anonymised data may be granted following review. Applications for access to data from the third round of the National Audit of Dementia should be made to the Healthcare Quality Improvement Partnership. Details of the process for obtaining these data are available at: https://www.hqip.org.uk/national-programmes/accessing-ncapop-data/#.W-7gS0ca7oo

For peer review only - http://bmjopen.bmj.com/site/about/guidelines.xhtml

Article Summary

Strengths and limitations of this study

- We used data from 98% of all acute hospitals in England and Wales, so our results are generalisable across the country.
- People with lived experience of dementia and caring for people with dementia helped us select items for including in the analysis and interpret the findings.
- All data were observational, so we cannot know whether the associations we found are causal.
- This research relied on audit data of variable quality. Data on length of stay and organisational checklist were complete.
- We did not have data on liaison psychiatric services in a sixth of hospitals, limiting our ability to explore whether the provision of these services influences the length of stay of people with dementia.

Funding statement

The work was supported by the Health Services and Delivery Research programme of the National Institute for Health Research (HS&DR 14/154/09). Infrastructure support for this research was provide by the NIHR Imperial Biomedical Research Centre (BRC).

Competing interests

Institutions for all authors have received funding from NIHR for other studies. Dr Alan Quirk and Ms Chloe Hood work at the Royal College of Psychiatrists and oversee the National Audit of Dementia. Professor Mike Crawford is Director of the College Centre for Quality Improvement at the Royal College of Psychiatrists and has been a member of the NIHR HTA General Committee. Professor Peter Crome was chair of the Steering Group for the National Audit of Dementia and has been on the HTA PCCPI Panel and HTA Prioritisation Committee A (Out of Hospital). Professor Kate Seers has been a member of the HS&DR Commissioning Board (Researcher Led). Professor Sophie Staniszewska has been on the following committees: HS&DR Associate Board Members; HS&DR Researcher Led - Associate Board Members; INVOLVE BOARD. All authors declare no other competing interests.

For peer review only - http://bmjopen.bmj.com/site/about/guidelines.xhtml

INTRODUCTION

The number of people with dementia who are admitted to acute hospitals is increasing.[1 2] The unfamiliar environment and disruption to daily routines that people with dementia experience when they are admitted to hospital can cause emotional distress and precipitate behavioural sypmotms.[3] When people with dementia are admitted to hospital they need more care and are more likely to suffer functional decline.[4] Patients with dementia are more impaired and vulnerable than patients without dementia and are at greater risk of adverse outcomes.[5] Compared to other inpatients, patients with dementia spend more time in hospital, are more likely to be readmitted within 28 days of their discharge,[6] and have significantly higher costs.[7 8]

A number of interventions have been developed to try to improve the quality of acute inpatient care for people with dementia. These efforts include the deployment of specialist nurses, the expansion of mental health liaison teams and the development of specialist inpatient units. A number of hospitals employ specialist dementia nurses to support the management of patients with dementia[9], but it is unclear whether this influences the amount of time that people with dementia spend in hospital.[10] Specialist units aim to better meet the needs of inpatients with dementia[11] by changing the environment of the ward, training staff to deliver patient centred care and delivering a programme of organised therapeutic and diversionary activities.[12] While carers of patients admitted to these units report improved satisfaction, there is no evidence to date that they impact on the amount of time that people stay in hospital.[13]

Tadros and colleagues reported that the implementation of a new integrated psychiatric liaison service reduced the amount of time that elderly patients admitted to City Hospital, Birmingham (UK), spent in hospital [14]. These findings prompted an expansion of mental health liaison services nationwide [15],but the findings of this study are yet to be replicated. Carer involvement has been identified as crucial to better patient experience during hospital admission. To achieve this, family carers need to be kept informed and provided with support and help when necessary.[16]

National audits examining acute care for people with dementia have highlighted considerable variation in the quality of care that hospitals deliver to people with dementia,[17 18] but little is known about reasons for these differences. Therefore, our objective was to identify aspects of the organisation and delivery of care for inpatients with dementia that are associated with shorter length of stay. This analysis was conducted as part of a wider study that examined the quality of care for inpatients with dementia.[19] As part of this mixed-methods study, we interviewed ward staff, managers and carers of people with dementia to explore factors that influence amount of time that people stay in hospital.

For peer review only - http://bmjopen.bmj.com/site/about/guidelines.xhtml

METHODS

Study setting and sample

We analysed data from a retrospective cohort of patients who were enrolled in the third round of National Audit of Dementia.[17] The audit aimed to evaluate the quality of care provided by all acute hospitals in England and Wales. We used data from three components of the audit: a hospital-level organisational checklist; a retrospective case note audit with a target of a minimum of 50 sets of patient notes of patients who had been given a clinical diagnosis of dementia and been admitted to hospital for 72 hours or longer between April 2016 to November 2016; and a survey of carer experience of quality of care. We combined these with the data from the second round of national survey of Liaison Psychiatry services in England, which collected data on mental health liaison service provision in all the acute hospitals in England.[20]

Study measures

We calculated the length of admission for each audit participant using their date of admission and date of discharge. To select independent variables for the analysis from the wide range included in the audit, we identified factors which may influence length of stay based on a scoping review of the literature.[19] We presented this list to the members of the Project Steering Group for their feedback, including people with lived experience of dementia and carers of people with dementia. In the final stage, we excluded any variables present in over 90% of hospitals. The lists of included variables and those excluded, and source of data are presented in online supplemental appendices 1 and 2.

Data management and analysis

Some of the predictor variables had multiple categories with small numbers in each category, we therefore combined these to obtain stable results [21] (details are available upon request). Primary diagnosis/cause of admission was taken as the first reason entered on the case note audit. Case notes included over 100 categories of primary diagnosis. We grouped these together in 11 categories such that myocardial infarction was combined with other vascular conditions and kidney and urological conditions were grouped together.

We conducted univariate analyses to explore the association between the dependent variable (length of stay) and patient-level (age, gender, primary diagnosis), ward-level (type of ward), and hospital-level (access to liaison mental health services, deployment of specialist dementia nurses, involvement of the Trust Board) predictor variables. We used Multiple Linear Regression for our analysis. Given the nested structure of the data, the final analysis was carried out using Hierarchical

For peer review only - http://bmjopen.bmj.com/site/about/guidelines.xhtml

Models. The main analysis was conducted on the complete data so missing data were not given any treatment. All data were analysed using statistical packages STATA (version 13) and SPSS 23.

The outcome measure, length of admission, was skewed so the logarithm of length of stay was used. We excluded all those who died during their admission from all analyses.

In order to test the robustness of associations found in the main analysis, we conducted two sensitivity analyses. In the first sensitivity analysis we restricted the samples to patients who were admitted with hip fractures/ trauma. In the second sensitivity analysis we restricted the sample to patients who were admitted to hospitals that scored above the median level of carer satisfaction in the carer survey that was conducted as part of the audit.[17] This was to address concerns raised by members of our Steering Group that hospitals could achieve shorter length of stay by prematurely discharging patients with dementia. We therefore conducted this sensitivity analysis to explore whether factors associated with shorter length of stay were not at the cost of lower levels of care satisfaction. Researchers who conducted the analysis of data were not involved in collecting and cleaning of data.

Ethical approval

We received approval for the secondary analysis of data from the National Clinical Audit and Patient Outcomes Programme from the Healthcare Quality Improvement Partnership prior to the start of study (Reference - HQIP162)

Patient and Public Involvement

We worked closely with the Steering Group for the National Audit of Dementia, which included people with lived experience of dementia and caring for people with dementia. In addition, the Project Management Group for the study included a member with dementia and a carer representative. Patients and carers contributed to the study by helping us select items for including in the analysis from the long list that we had originally developed. They also contributed to interpretation of study findings. Some of the patients involved in the early stages of the study were not able to contribute to the later stages due to the progression of their condition. One of the carer representatives (G.Z.), helped us prepare this manuscript and write the lay summary for the study.

RESULTS

In total, 200 (98.5%) of 203 acute hospitals in England and Wales took part in the audit. Organisational checklists were returned from all 200 hospitals along with data on 10,106 patient records and 4,688 carer questionnaires. The mean age of participants was 84.3 (SD=7.9), 8274

For peer review only - http://bmjopen.bmj.com/site/about/guidelines.xhtml

(81.9%) were White, and 6054 (59.9%) were female. Descriptive data on the predictor variables from the audit are presented in online supplemental appendices 3, 4 and 5.

Liaison Psychiatry Data

Data for the 2015 audit of psychiatric liaison services were returned by teams serving 176 (88%) of the 200 hospitals that took part in the national audit of dementia. We used data on the two predictor variables in our analysis; the number of hours covered by the liaison service, and whether the team included an old age psychiatrist. Ninety-one (53.53%) liaison services had 24-hour coverage, 72 (42.35%) were available for more than 40 hours per week, and 4 (2.35%) for less than 40 hours per week. In total, 83 of 169 (49.11%) liaison services included an old age psychiatrist. Full details can be found in online supplemental appendix 6.

Length of stay

Valid data on the length of stay were available for 10,105 patients with dementia; the median length of stay was 12 days (interquartile range = 6 to 23 days). There was variation in the median length of stay across the 200 hospitals, ranging from five to 39 days (interquartile range = 10 to 14 days).

The results of the multivariate analyses are presented in table 1. The following variables were associated with shorter average length of hospital stay: presenting with a respiratory condition, Black, Asian and Minority Ethnic background, initiating the discharge planning within 24 hours of admission, and patients for whom there was not a record of discussion with the responsible consultant (only in hospitals with a specialist old age psychiatrist). In hospitals where Trust Boards regularly review delayed discharges, there was a non-statistically significant trend towards patients having shorter hospital stays.

Variables associated with longer average length of stay were; type of ward that patients were being treated on with those on the care of the elderly having longer average hospital stays compared to general medical and surgical wards, and discussions with carers being recorded in patients' case notes.

All risk factors were included in the initial full-effects model. This model was then reduced by excluding stepwise non-significant effects. The model presented here is the model with only the significant effects.

For peer review only - http://bmjopen.bmj.com/site/about/guidelines.xhtml

Table 1 - Multivariate analysis of factors associated with length of stay among 8,817 patients with dementia treated at 196 acute hospitals

Predictor variable	Interactions*	Estimated effect (95% CI)	p value
**Patient age**		-0.003 (-0.005 to 2.19)	0.052
**Patient ethnicity** – White		-	
Black, Asian and Minority Ethnic		-0.066 (-0.13 to -0.002)	0.043
**Ward type** - Care of the elderly		-	
Cardiac		-0.33 (-0.47 to -0.19)	< 0.001
General Medical		-0.28 (-0.33 to -0.22)	< 0.001
Orthopaedics		-0.16 (-0.26 to -0.05)	0.003
Surgical		-0.32 (-0.41 to -0.23)	< 0.001
Other medical		-0.26 (-0.33 to -0.18)	< 0.001
Other		-0.56 (-0.74 to -0.37)	< 0.001
**Primary Diagnosis -** Respiratory		-	
Fall		0.24 (0.17 to 0.32)	< 0.001
Orthopaedic		0.39 (0.27 to 0.50)	< 0.001
Delirium/Confusion		0.25 (0.18 to 0.33)	< 0.001
Other		0.13 (0.05 to 0.20)	0.001
**Discharge planning within 24 hours of admission** *Yes Vs No*		-0.24 (-0.29 to -0.18)	< 0.001
**Executive Board reviews delayed discharge** *Yes Vs No*		-0.08 (-0.17 to 0.006)	0.069**
**Evidence of discussing discharge with carers** *Yes Vs No*		0.26 (0.21 to 0.32)	< 0.001
**Evidence of discussing discharge with consultant**	**In hospitals with old age liaison psychiatrist *Yes vs No***		
Yes		-0.039 (-0.12 to 0.05)	0.367
No		-0.15 (-0.03 to -0.26)	0.012

* When an interaction effect is present the estimated effects of risk factors are interpreted taking into effect the interaction column

For peer review only - http://bmjopen.bmj.com/site/about/guidelines.xhtml

For peer review only

Sensitivity analyses

Results from two sensitivity analyses are presented in online supplemental appendices 7 and 8. We looked at factors associated with length of stay among 669 patients treated for hip fracture at 170 acute hospitals. We also performed multivariate analysis of factors associated with length of stay among 3375 patients treated at 74 acute hospitals with higher carer-rated satisfaction. There were fewer associations in the first sensitivity analysis when we restricted the sample to patients who were admitted to hospital due to hip fracture or related injuries (n=669). The variables associated with shorter average length of stay were: documented evidence of initiating discharge planning within 24 hours of admission, Black, Asian and Minority Ethnic background (only among women but not men), and female patients in those hospitals that deployed dementia nurse specialists. When there was documented evidence that discharge had been discussed with carers, patients had longer hospital stays.

In the analysis of data from the sub-sample of patients admitted to the 74 hospitals that had carer-rated satisfaction scores above the median (n=3375), primary diagnosis and type of ward were still associated with length of stay. Patients with respiratory conditions had shorter hospital admissions compared to other diagnoses, including cardiac/vascular, fall and hip fracture. Those on the care of the elderly wards seemed to have longer lengths of stay compared to patients being treated on general medical, cardiac, orthopaedics, and surgical wards. When there was no documented evidence of discussing discharge with carers, female patients had shorter hospital admissions. In hospitals where the liaison service included an old age specialist consultant psychiatrist, patients had shorter hospital stays when there was evidence that discharge planning had been initiated within the first 24 hours of admission, as well as in hospitals where there were not any social workers or designated persons. In situations when there was evidence of discussing discharge with the consultant responsible for patient's care, average length of stay was higher. Table 2 presents a summary of results from the main multivariate and two sensitivity analyses.

For peer review only - http://bmjopen.bmj.com/site/about/guidelines.xhtml

Table 2 – Summary of results from the main multivariate and two sensitivity analyses of modifiable predictor variables

		Main multivariate analysis	Admitted with hip fracture (n = 669)	Admitted to hospitals with higher carer-satisfaction (n = 3375)
**Predictor variable**	**Interactions***	**Estimated effect (95% CI) P value**		
**Discharge planning within 24 hours of admission** *Yes Vs No*		-0.24 (-0.29 to -0.18) < 0.001	-0.38 (-0.55 to -0.21) < 0.001	
Yes	**Old age liaison psychiatrist consultant** ***Yes vs No***			-0.29 (-0.46 to -0.11) 0.002
No				-0.07 (-0.25 to 0.11) 0.425
**Evidence of discussing discharge with carers** *Yes Vs No*		0.26 (0.21 to 0.32) < 0.001	0.41 (0.27 to 0.55) < 0.001	
Yes	***Gender of the patient*** *Female vs male*			0.005 (-0.07 to 0.08) 0.901
No				-0.20 (-0.34 to -0.05) 0.008

* When an interaction effect is present the estimated effects of risk factors are interpreted taking into effect the interaction column

For peer review only - http://bmjopen.bmj.com/site/about/guidelines.xhtml

For peer review only

DISCUSSION

We aimed to identify measures that are associated with shorter hospital admissions for patients with dementia. In the multivariate analysis, in addition to primary diagnosis and the type of ward that patients were admitted to, we found that the ethnicity, discharge planning within 24 hours of admission, and documented discussions about discharge with carers were associated with the amount of time that patients spent in hospital. Whilst presenting with a respiratory condition, BAME background, and early discharge planning were associated with shorter hospital stays, documented discussions with carers was associated with longer average hospital stays. These associations were supported by sensitivity analysis of patients admitted with a hip fracture and in hospitals with higher carer-rated quality of care for people with dementia.

Of the modifiable variables, planning discharge within 24 hours of admission had the strongest negative association with length of hospital stay (Co-efficient =-0.24).

These data support those of a smaller scale study of 85 patients who spent more than 14 days in an acute hospital in Ireland, which identified timely discharge planning and clear communication as measures for preventing prolonged hospital stays. [22] Support for early discharge planning also comes from the results of a series of case studies conducted by NHS trusts across England as part of their quality improvement programmes (https://improvement.nhs.uk/resources/guide-reducing-long-hospital-stays/).

Patients with dementia are at increased risk of mortality and morbidity when hospitalised, and health professionals are usually faced with high levels of complex care needs when managing care and discharge of this patient group. [23] In a literature review of barriers and facilitators to discharge planning for people with dementia, involving family carers in the early stages of admission was identified to improve discharge process.[24]

In the wider mixed methods study, we carried out interviews with clinicians and senior managers to shed light on the associations found in quantitative data analysis. In these interviews, we found discharge planning among patients with dementia is not straightforward. Staff told us that despite the complexity, setting a planned discharge date as soon as preliminary investigations are carried out and working out the potential barriers to a timely discharge are important.[19] Senior nurses told us that allocating tasks to specific members of staff and daily review of the progress would ensure that problems are identified early and escalated to relevant departments.

In our interviews we also explored the association found between carer involvement and longer admissions. There was a consensus that involving family carers helps patients settle and adhere with

For peer review only - http://bmjopen.bmj.com/site/about/guidelines.xhtml

treatments when admitted to hospital. Family carers usually acknowledge that long hospital stays are not conducive to patients' wellbeing. However, lack of clear communication between family carers and hospital staff from the outset may mean that expectations are not shared, which might lead to longer admissions to hospital. In a previous literature review of barriers and facilitators to discharge planning for people with dementia, researchers identified involving family carers in the early stages of admission to improve discharge process.[24]

In our univariate analysis, we found that people admitted to specialist care of the elderly wards spent longer in hospital, than those admitted to general medical and surgical wards. However, ward type did not predict length of stay once the effect of demographic and clinical factors was taken into account in the multivariate analysis. These findings may reflect the complex mental, physical, and social needs of people admitted to specialist wards. [25]

The association between Black, Asian and Minority Ethnic background and shorter length of stay was unexpected and require further investigation.

Strengths and weaknesses of the study

We used data from the third round of the National Audit of Dementia from 98% of all acute hospitals in England and Wales. This means that our results could be generalisable across the country. Data was complete on length of stay, and level of data completeness was high for key variables.

In this study we had the advantage of involvement of people with lived experience of dementia and carers of people with dementia in various stages of study planning, data analysis and interpretation. The study has several limitations. We relied on the information submitted to the audit team by hospital staff on whether hospitals had adopted a strategy or not. We cannot rule out the possibility that in some instances, certain assessments or discussions might have taken place but not been recorded. Whilst the large dataset makes the findings more generalisable, we cannot be certain if the associations we found are causal. For instance, our finding that patients whose records included documented evidence of discussions with carers having longer admissions could be causal i.e. that discussions with carers raised additional issues that they felt needed to be addressed before the person they cared for was discharged home. However, it could also be confounded by complexity and co-existing conditions of patients i.e. staff needed to liaise more with carers of patients with complex conditions, and such patients also required a longer period of treatment in hospital. It is possible that documenting discussions with carers happens more often in complex cases, where there has been more deterioration, or there is need for change of residence. [26]

For peer review only - http://bmjopen.bmj.com/site/about/guidelines.xhtml

There can be a trade-off between shorter hospital stay and other aspects of quality of care. Our finding that the association between shorter length of stay and early discharge planning was also present in hospitals with higher carer-rated quality of care was reassuring.

Our findings reflect practice from 2016. Since these data were collected, the COVID pandemic has further highlighted the importance of discharge planning to ensure that patient safety is maintained, and premature discharge avoided. Discharge planning at the time of COVID pandemic has been even more complicated as care homes might be unwilling to have patients back immediately after discharge from acute hospitals. Concerns about COVID outbreaks among care home residents mean that family carers are reluctant for their loved ones to return to care homes. [27 28]

Previous research has raised the possibility that psychiatric liaison services might enable more timely discharge of older adults with mental health conditions including dementia.[14 29] Missing data on availability of psychiatric liaison services meant that we could not fully examine the potential impact of this factor on length of stay.

Implications for practice and future research

We recommend that hospitals put systems in place for planning discharge as soon as possible regardless of the complexity of patient presentation. Clear communication with family carers from early stages of admission helps them form realistic expectations to avoid unnecessary delays in discharge.

Future research should explore the unexpected finding of shorter hospital stays among patients with dementia of from Black Asian and Minority Ethnic backgrounds.

Contribution of authors

RS was the lead researcher, assisted with the analysis of quantitative data, and prepared the final manuscript. AB advised on the methods. PC chaired the Steering Group and contributed to the interpretation of the results. FG led the analysis of quantitative data. CH advised on the methods and contributed to the interpretation of the results. WL provided advice and support on the analysis of data from the second national survey of Mental Health Liaison Services. AQ contributed to the interpretation of the results. KS contributed to the interpretation of the results. SS contributed to the interpretation of the results. GZ contributed to the interpretation of the results. MJC was the

For peer review only - http://bmjopen.bmj.com/site/about/guidelines.xhtml

chief investigator and oversaw all aspects of the study. All authors read, made comments and approved the final manuscript.

Acknowledgements

We acknowledge the valuable contribution made by our collaborators: Daphne Wallace (retired psychiatrists with lived experience), Amy Claringbold (Trial manager, Imperial College London), and Sara Hammond who led the scoping literature review and helped with the interpretation of the results.

We would also like to thank patients and carers and other members of the Steering Group, including: Nicci Gerrard (Carer Representative, John's Campaign), Sue Pierlejewski (Carer Representative), Hilary Doxford (Alzheimer's Society Ambassador), Jayne Goodrick (Carer Representative) Chris Roberts (Alzheimer's Society Ambassador)

For peer review only - http://bmjopen.bmj.com/site/about/guidelines.xhtml

REFERENCES

1. Ferri CP, Prince M, Brayne C, et al. Global prevalence of dementia: a Delphi consensus study. *Lancet* 2005;366(9503):2112-7. doi: 10.1016/S0140-6736(05)67889-0
2. Ombudsman HS. Care and compassion? Report of the health service ombudsman on ten investigations into NHS care of older people. London: The Stationery Office, 2011.
3. Porock D, Clissett P, Harwood RH, et al. Disruption, control and coping: responses of and to the person with dementia in hospital. *Ageing Soc* 2015;35(1):37-63. doi: 10.1017/s0144686x13000561 [published Online First: 2015/04/17]
4. Mukadam N, Sampson EL. A systematic review of the prevalence, associations and outcomes of dementia in older general hospital inpatients. *International psychogeriatrics* 2011;23(3):344-55.
5. Travers C, Byrne GJ, Pachana NA, et al. Prospective observational study of dementia in older patients admitted to acute hospitals. *Australasian journal on ageing* 2014;33(1):55-58.
6. Tropea J, LoGiudice D, Liew D, et al. Poorer outcomes and greater healthcare costs for hospitalised older people with dementia and delirium: a retrospective cohort study. *International journal of geriatric psychiatry* 2017;32(5):539-47.
7. Connolly S, O'Shea E. The impact of dementia on length of stay in acute hospitals in Ireland. *Dementia* 2015;14(5):650-58. doi: 10.1177/1471301213506922
8. Alzheimer's Society. Counting the cost. Caring for people with dementia on hospital wards. London: Alzheimer's Society 2009.
9. Elliot R, Adams J. The creation of a Dementia Nurse Specialist role in an acute general hospital. *J Psychiatr Ment Health Nurs* 2011;18(7):648-52. doi: 10.1111/j.1365-2850.2011.01771.x
10. Griffiths P, Bridges J, Sheldon H, et al. The role of the dementia specialist nurse in acute care: a scoping review. *Journal of clinical nursing* 2015;24(9-10):1394-405.
11. Harwood RH, Porock D, King N, et al. Development of a specialist medical and mental health unit for older people in an acute general hospital. Nottingham: University of Nottingham, 2010.
12. Goldberg SE, Bradshaw LE, Kearney FC, et al. Care in specialist medical and mental health unit compared with standard care for older people with cognitive impairment admitted to general hospital: randomised controlled trial (NIHR TEAM trial). *BMJ* 2013;347:f4132. doi: 10.1136/bmj.f4132
13. Lai C, Yeung J, Mok V, et al. Special care units for dementia individuals with behavioural problems. . CD006470: Cochrane Database of Systematic Reviews. No. 4, 2009.
14. Tadros G, Salama RA, Kingston P, et al. Impact of an integrated rapid response psychiatric liaison team on quality improvement and cost savings: the Birmingham RAID model. *The Psychiatrist* 2013;37(1):4-10.
15. House A, West R, Smith C, et al. The effect of a hospital liaison psychiatry service on inpatient lengths of stay: interrupted time series analysis using routinely collected NHS hospital episode statistics. *BMC psychiatry* 2020;20(1):27.
16. Beardon S, Patel K, Davies B, et al. Informal carers' perspectives on the delivery of acute hospital care for patients with dementia: a systematic review. *BMC geriatrics* 2018;18(1):23. doi: 10.1186/s12877-018-0710-x [published Online First: 2018/01/27]
17. Royal College of Psychiatrists. National Audit of Dementia care in general hospitals 2016-2017: Third round of audit report.: Royal College of Psychiatrists, 2017.
18. Young J, Hood C, Gandesha A, et al. National Audit of Dementia care in general hospitals 2012–13: Second round audit report and update. London: Royal College of Psychiatrists, Healthcare Quality Improvement Partnership, 2013.
19. Sanatinia R, Crawford MJ, Quirk A, et al. Identifying features associated with higher-quality hospital care and shorter length of admission for people with dementia: a mixed-methods study. *Health Services and Delivery Research* 2020;8:1-92. doi: 10.3310/hsdr08220

For peer review only - http://bmjopen.bmj.com/site/about/guidelines.xhtml

20. Barrett J, Aitken P, Lee W. Report of the 2nd Annual Survey of Liaison Psychiatry in England. In: Liaison RotnASo, England. Pi, eds. London: National Collaborating Centre for Mental Health, 2015.
21. Barkan H. Statistics in clinical research: Important considerations. *Annals of Cardiac Anaesthesia* 2015;18(1):74.
22. Short E, D'Alton M, Dolan E, et al. 121 Patients with Prolonged Lengths of Stay. A Point Prevalence Study. *Age & Ageing* 2019;48
23. Fox A, MacAndrew M, Ramis MA. Health outcomes of patients with dementia in acute care settings—A systematic review. *International journal of older people nursing* 2020;15(3):e12315.
24. Stockwell-Smith G, Moyle W, Marshall AP, et al. Hospital discharge processes involving older adults living with dementia: An integrated literature review. *Journal of clinical nursing* 2018;27(5-6):e712-e25. doi: 10.1111/jocn.14144
25. Alabaf Sabbaghi S, De Souza D, Sarikonda P, et al. Allocating patients to geriatric medicine wards in a tertiary university hospital in England: A service evaluation of the Specialist Advice for the Frail Elderly (SAFE) team. *Aging Medicine* 2018;1(2):120-24.
26. Timmons S, O'Shea E, O'Neill D, et al. Acute hospital dementia care: results from a national audit. *BMC geriatrics* 2016;16(1):113. doi: 10.1186/s12877-016-0293-3
27. Mok VCT, Pendlebury S, Wong A, et al. Tackling challenges in care of Alzheimer's disease and other dementias amid the COVID-19 pandemic, now and in the future. *Alzheimers Dement* 2020:10.1002/alz.12143. doi: 10.1002/alz.12143
28. Carter R. Covid-19: The support UK care homes need to survive. *BMJ* 2020;369:m1858. doi: 10.1136/bmj.m1858
29. Singh I, Ramakrishna S, Williamson K. The Rapid Assessment Interface and Discharge service and its implications for patients with dementia. *Clinical interventions in aging* 2013;8:1101-8. doi: 10.2147/CIA.S36398

For peer review only - http://bmjopen.bmj.com/site/about/guidelines.xhtml

Appendix 1- Predictor variables included in the analysis of factors associated with length of admission

Predictor variable	Source of data	Audit item
Type of ward	Case note audit	Q5
Primary Diagnosis	Case note audit	Q6
Discharge planning within 24 hours of admission	Case note audit	Q34
Evidence of discussing discharge with carers	Case note audit	Q29b
Executive board reviews delayed discharge	Organisational checklist	Q2b
Evidence of discussing discharge with consultant responsible for the patient	Case note audit	Q29c
Dementia specialist nurse	Organisational checklist	Q6
Social worker, or other designated person	Organisational checklist	Q34
Dementia care pathway/bundle	Organisational checklist	Q1
Liaison Hours	Liaison Psychiatry Survey of England 2	Q21
Older adult consultant	Liaison Psychiatry Survey of England 2	Q14
**Demographic variables**		
Age of the patient	Case note audit	Q1
Gender of the patient	Case note audit	Q2
Ethnicity of the patient	Case note audit	Q3

Appendix 2- List of predictor variables excluded from the analysis because over 90% of hospitals reported having implemented them.

Predictor variable	Source of data	Audit question number	Proportion of hospitals reporting them (%)
Named person/identified team with overall responsibility for complex needs discharge	Organisational checklist	Q32	95.5%
Access to intermediate care for people with dementia	Organisational checklist	Q30	92.5%

For peer review only

Dementia working group in place	Organisational checklist	Q9	93.5%
Evidence-based tool is used for establishing ward staffing levels	Organisational checklist	Q11	98.5%
Dementia champion at ward level	Organisational checklist	Q5a	93.5%

Appendix 3 -Number and proportion of hospitals with policies and practices in place aimed at supporting effective care for people with dementia

**Predictor variable**	**Number**	**Percentage**
Executive board reviews delayed discharge		
Yes	63	31.5%
No	137	68.5%
Dementia specialist nurse*		
Yes	64	32%
No	136	68%
Dementia care pathway/bundle		
Yes	121	60.5%
No	26	13%
In development	53	20.5%
Social worker, or other designated person		
Yes	152	76%
No	48	24%

Appendix 4 - Descriptive statistics of the predictor variables used from case note audit questionnaire (N=10106)

**Predictor variable**	**Number**	**Percentage (SD)**
**Age** (N= 10096)<br>Mean (SD)	84.3	(7.9)

For peer review only

**Gender**		
Male	4052	40.1%
Female	6054	59.9%
**Ethnicity**		
White	8274	81.9%
Black, Asian and Minority Ethnic	1622	16%
Not documented	210	2.1%
**Specialty of the ward**		
Cardiac	248	2.5%
Care of the elderly/complex care	4125	40.8%
Critical Care	23	0.2%
General Medical	2397	23.7%
Nephrology	52	0.5%
Obstetrics/Gynaecology	41	0.4%
Oncology	22	0.2%
Orthopaedics	906	9.0%
Stroke	457	4.5%
Surgical	686	6.8%
Other medical	1000	9.9%
Other	136	1.3%
Unknown	13	0.1%
**Primary diagnosis (N=10048)**		
Respiratory	2005	19.8
Fall	1346	13.3
Urinary/Renal	906	9.0
Hip fracture/dislocation/ other fractures/trauma	886	8.8
Sepsis	635	6.3
Delirium/Confusion/ Cognitive Impairment	1204	11.9
Gastrointestinal	595	5.9
Cardiac/ vascular / chest pain	518	5.1
Stroke + Neurological	750	7.4
Other	1239	12.3
Missing	22	0.2

Appendix 5- Number and proportion of patients receiving care according to audit standards (N=10106)

Predictor variable	Number	Percentage
**Evidence of discussing discharge with carer**		
Yes	5628	55.7%
No	1359	13.4%
N/A	398	3.9%
Missing	2721	26.9%
**Evidence of discussing discharge with consultant**		
Yes	5529	54.7%
No	1856	25.1%
Missing	2721	26.9%

For peer review only

Discharge planning initiated within 24 hours of admission		
Yes	2499	24.7%
No	2791	27.6%
N/A	2095	20.7%
Missing	2721	26.9%

Appendix 6- Provision of psychiatric liaison services at 200 hospitals

Predictor variable	Number	Percentage
**Liaison hours**		
No service	3	1.5%
Part-time (less than 40 hours per week)	1	0.5%
Working hours (40 hours per week)	3	1.5%
Extended Hours (More than 40 hours per week)	72	36.0%
24 Hours	91	45.5%
Missing	30	15.0%
**Team includes a specialist old age psychiatrist**		
Yes	83	41.5%
No	86	43.0%
Missing	31	15.5%

Appendix 7 -Multivariate analysis of factors associated with length of stay among 669 patients treated for hip trauma/ fracture at 170 acute hospitals

Predictor variable	* Interactions	Estimated effect (95% CI)	p value
**Ward Type** - *Care of the elderly*		-	
Orthopaedics		0.46 (0.25 to 0.66)	< 0.001
Surgical		0.34 (0.04 to 0.65)	0.29
Other medical		Ns	
Other (non-medical)		1.34 (0.31 to 2.36)	0.011
**Executive Board reviews delayed discharge**	Age		
Yes		-0.02 (-0.04 to -0.005)	0.010
No		0.01 (-0.00 to 0.20)	0.102
**Evidence of discussing discharge with carers** *Yes Vs No*		0.41 (0.27 to 0.55)	< 0.001

For peer review only

**Discharge planning within 24 hours of admission** *Yes Vs No*		-0.38 (-0.55 to -0.21)	< 0.001
**Ethnicity**	**Gender** *Female VS Male*		
BAME		-0.51 (-0.85 to -0.18)	0.003
White		-0.06 (-0.23 to 0.11)	0.474
**Dementia specialist nurse**	**Gender** *Female VS Male*		
Yes		-0.47 (-0.75 to -0.20)	0.001
No		-0.11 (-0.32 to 0.11)	0.33

* When an interaction effect is present the estimated effects of risk factors are interpreted taking into effect the interaction column

Appendix 8 -Multivariate analysis of factors associated with length of stay among 3375 patients treated at 74 acute hospitals with higher carer-rated satisfaction

Predictor variable	*Interactions	Estimated effect (95% CI)	p value
**Ward Type -** Care of the elderly		-	
Cardiac		-0.40 (-0.64 to -0.16)	0.001
General Medical		-0.28 (-0.38 to -0.19)	< 0.001
Orthopaedics		-0.23 (-0.40 to -0.06)	0.008
Surgical		-0.39 (-0.53 to -0.25)	< 0.001
Other medical		-0.37 (-0.49 to -0.25)	< 0.001
Other (non-medical)		-0.57 (-0.86 to -0.28)	< 0.001
**Primary Diagnosis -** Respiratory		-	
Fall		0.30 (0.18 to 0.41)	< 0.001
Hip fracture/trauma		0.50 0.31 to 0.67	< 0.001
Cardiac/Vascular		0.21 (0.04 to 0.39)	0.019
Delirium/Confusion		0.31 (0.18 to 0.43)	< 0.001
Other		0.18 (0.05 to 0.30)	0.006
**Evidence of discussing discharge with carers**	***Gender of the patient*** *Female vs male*		

For peer review only

Yes		0.005 (-0.07 to 0.08)	0.901
No		-0.20 (-0.34 to -0.05)	0.008
**Discharge planning within 24 hours of admission**	**OA liaison psychiatrist consultant** *Yes vs No*		
Yes		-0.29 -0.46 to -0.11	0.002
No		-0.07 (-0.25 to 0.11)	0.425
**Social worker or other designated person**	**OA liaison psychiatrist consultant** Yes *vs No*		
Yes		-0.04 (-0.19 to 0.12)	0.645
No		-0.41 (-0.66 to -0.16)	0.002
**Evidence of discussing discharge with consultant**** *Yes vs No*		0.13 (0.04 to 0.22)	0.005

* When an interaction effect is present the estimated effects of risk factors are interpreted taking into effect the interaction column

** Evidence in the notes that the discharge coordinator/person or team planning discharge has discussed place of discharge and support needs with the consultant responsible for the patient's care. This can be together as a summary or recorded as separate discussions.

For peer review only

Reporting checklist for cohort study.

Based on the STROBE cohort guidelines.

Instructions to authors

Complete this checklist by entering the page numbers from your manuscript where readers will find each of the items listed below.

Your article may not currently address all the items on the checklist. Please modify your text to include the missing information. If you are certain that an item does not apply, please write "n/a" and provide a short explanation.

Upload your completed checklist as an extra file when you submit to a journal.

In your methods section, say that you used the STROBE cohortreporting guidelines, and cite them as:

von Elm E, Altman DG, Egger M, Pocock SJ, Gotzsche PC, Vandenbroucke JP. The Strengthening the Reporting of Observational Studies in Epidemiology (STROBE) Statement: guidelines for reporting observational studies.

		Reporting Item	Page Number
**Title and abstract**			
Title	#1a	Indicate the study's design with a commonly used term in the title or the abstract	1, 2
Abstract	#1b	Provide in the abstract an informative and balanced summary of what was done and what was found	2

For peer review only

**Introduction**			
Background / rationale	#2	Explain the scientific background and rationale for the investigation being reported	4
Objectives	#3	State specific objectives, including any prespecified hypotheses	4
**Methods**			
Study design	#4	Present key elements of study design early in the paper	5
Setting	#5	Describe the setting, locations, and relevant dates, including periods of recruitment, exposure, follow-up, and data collection	5
Eligibility criteria	#6a	Give the eligibility criteria, and the sources and methods of selection of participants. Describe methods of follow-up.	5
Eligibility criteria	#6b	For matched studies, give matching criteria and number of exposed and unexposed	N/A
Variables	#7	Clearly define all outcomes, exposures, predictors, potential confounders, and effect modifiers. Give diagnostic criteria, if applicable	5, Appendices 1 and 2
Data sources / measurement	#8	For each variable of interest give sources of data and details of methods of assessment (measurement). Describe comparability of assessment methods if there is more than one group. Give information separately for for exposed and unexposed groups if applicable.	5

For peer review only

Bias	#9	Describe any efforts to address potential sources of bias	5
Study size	#10	Explain how the study size was arrived at	N/A
Quantitative variables	#11	Explain how quantitative variables were handled in the analyses. If applicable, describe which groupings were chosen, and why	5
Statistical methods	#12a	Describe all statistical methods, including those used to control for confounding	5,6
Statistical methods	#12b	Describe any methods used to examine subgroups and interactions	6
Statistical methods	#12c	Explain how missing data were addressed	6
Statistical methods	#12d	If applicable, explain how loss to follow-up was addressed	N/A
Statistical methods	#12e	Describe any sensitivity analyses	6
**Results**			
Participants	#13a	Report numbers of individuals at each stage of study—eg numbers potentially eligible, examined for eligibility, confirmed eligible, included in the study, completing follow-up, and analysed. Give information separately for for exposed and unexposed groups if applicable.	N/A, secondary analysis
Participants	#13b	Give reasons for non-participation at each stage	N/A

For peer review only

Participants	#13c	Consider use of a flow diagram	N/A
Descriptive data	#14a	Give characteristics of study participants (eg demographic, clinical, social) and information on exposures and potential confounders. Give information separately for exposed and unexposed groups if applicable.	6, tables and appendices
Descriptive data	#14b	Indicate number of participants with missing data for each variable of interest	6
Descriptive data	#14c	Summarise follow-up time (eg, average and total amount)	N/A
Outcome data	#15	Report numbers of outcome events or summary measures over time. Give information separately for exposed and unexposed groups if applicable.	N/A
Main results	#16a	Give unadjusted estimates and, if applicable, confounder-adjusted estimates and their precision (eg, 95% confidence interval). Make clear which confounders were adjusted for and why they were included	7
Main results	#16b	Report category boundaries when continuous variables were categorized	7
Main results	#16c	If relevant, consider translating estimates of relative risk into absolute risk for a meaningful time period	7
Other analyses	#17	Report other analyses done—e.g., analyses of subgroups and interactions, and sensitivity analyses	9

Discussion

For peer review only

Key results	#18	Summarise key results with reference to study objectives	11
Limitations	#19	Discuss limitations of the study, taking into account sources of potential bias or imprecision. Discuss both direction and magnitude of any potential bias.	12
Interpretation	#20	Give a cautious overall interpretation considering objectives, limitations, multiplicity of analyses, results from similar studies, and other relevant evidence.	11. 12, 13
Generalisability	#21	Discuss the generalisability (external validity) of the study results	12
**Other Information**			
Funding	#22	Give the source of funding and the role of the funders for the present study and, if applicable, for the original study on which the present article is based	3

Notes:

- 13a: N/A, secondary analysis
- 14a: 6, and tables and appendices The STROBE checklist is distributed under the terms of the Creative Commons Attribution License CC-BY. This checklist was completed on 17. November 2020 using https://www.goodreports.org/, a tool made by the EQUATOR Network in collaboration with Penelope.ai

For peer review only
